# Investigation of Thermal and Thermomechanical Properties of Biodegradable PLA/PBSA Composites Processed via Supercritical Fluid-Assisted Foam Injection Molding

**DOI:** 10.3390/polym9010022

**Published:** 2017-01-09

**Authors:** Sai Aditya Pradeep, Hrishikesh Kharbas, Lih-Sheng Turng, Abraham Avalos, Joseph G. Lawrence, Srikanth Pilla

**Affiliations:** 1 Department of Automotive Engineering, Clemson University, Clemson, SC 29607, USA; spradee@g.clemson.edu; 2 Department of Material Science and Engineering, Clemson University, Clemson, SC 29634, USA; 3 Polymer Engineering Center, Department of Mechanical Engineering, University of Wisconsin-Madison, Madison, WI 53706, USA; kharbas@gmail.com (H.K.); turng@engr.wisc.edu (L.-S.T.); 4 Polymer Institute, University of Toledo, Toledo, OH 43606, USA; Abraham.Avalos@utoledo.edu (A.A.); joseph.lawrence@utoledo.edu (J.G.L.)

**Keywords:** polylactide, poly(butylene succinate-co-adipate), compatibilization, crystallization, foaming

## Abstract

Bio-based polymer foams have been gaining immense attention in recent years due to their positive contribution towards reducing the global carbon footprint, lightweighting, and enhancing sustainability. Currently, polylactic acid (PLA) remains the most abundant commercially consumed biopolymer, but suffers from major drawbacks such as slow crystallization rate and poor melt processability. However, blending of PLA with a secondary polymer would enhance the crystallization rate and the thermal properties based on their compatibility. This study investigates the physical and compatibilized blends of PLA/poly (butylene succinate-co-adipate) (PBSA) processed via supercritical fluid-assisted (ScF) injection molding technology using nitrogen (N_2_) as a facile physical blowing agent. Furthermore, this study aims at understanding the effect of blending and ScF foaming of PLA/PBSA on crystallinity, melting, and viscoelastic behavior. Results show that compatibilization, upon addition of triphenyl phosphite (TPP), led to an increase in molecular weight and a shift in melting temperature. Additionally, the glass transition temperature (*T*_g_) obtained from the tanδ curve was observed to be in agreement with the *T*_g_ value predicted by the Gordon–Taylor equation, further confirming the compatibility of PLA and PBSA. The compatibilization of ScF-foamed PLA–PBSA was found to have an increased crystallinity and storage modulus compared to their physically foamed counterparts.

## 1. Introduction

Thermoplastic foams, as lightweight materials, are extensively used in sectors such as automotive, packaging, and aerospace due to advantages such as high strength-to-weight ratios, acoustic properties, low susceptibility to water vapor, superior impact resistance, and low densities [1]. However, a majority of these foams have precursors that are sourced from crude oil, which is a finite, non-renewable resource and a major cause of increasing carbon emissions that contribute to anthropogenic climate change. In the present paradigm, bio-based compostable thermoplastic foams have been gaining ground in many industries as they help to meet environmental regulations and standards set by international and domestic agencies via their application. Polylactic acid (PLA) is an aliphatic polyester that has emerged as one of the most commercially successful biopolymers due to its transparency, high strength, and high stiffness, making it superior to many other bio-based polymers [2,3,4,5]. However, despite its several advantages, commercially available PLA has many inherent weaknesses—in particular, its low toughness, low heat resistance, and brittleness—that prevent it from being widely adopted for durable applications. More importantly, PLA has poor melt processability due to a narrow processing window and a very slow crystallization rate. Typically, a higher crystallinity is desirable in finished products due to its strong influence on mechanical and thermal properties.

Several strategies exist to overcome the slow crystallization rate and melt processability of PLA, such as the addition of fillers [6,7], copolymerization [8,9], and melt-blending [10,11,12], all of which offer an effective medium for enhancing its overall performance. Among these, blending with inherently toughened (bio)polymers is one of the most effective solutions. However, most of these physical blends are immiscible in nature and can lead to the overall deterioration of properties [13]. The successful application of the reactive compatibilization technique has provided enormous opportunities to enhance the compatibility of blends that are otherwise immiscible and incompatible. Reactive compatibilization can be achieved via melt-blending of PLA with other suitable polymers, resulting in the formation of a block or graft copolymer at the interface and reducing the interfacial tension of immiscible polymer components, thereby promoting interfacial adhesion [13]. While blending PLA with toughened polymers enables us to overcome some of its aforementioned drawbacks, the foaming of PLA and its blends is critical to obtaining lightweight, sustainable thermoplastic foams.

In this context, supercritical fluid (ScF)-assisted injection molding, also known as microcellular injection molding, has been shown to broaden the processing window for biopolymers such as PLA, as it employs supercritical N_2_ or CO_2_ [14,15]. The broadening of the processing window is because ScF lowers the melt viscosity of the polymer [16,17] due to the formation of a single-phase polymer/gas solution, enabling the polymer to be processed at lower temperatures [18]. In addition ScF-assisted injection molding produces foamed components containing micron-sized cells and high cell densities while consuming a smaller amount of material and energy and having lower cycle times vs. conventional injection molding [19,20,21]. Hence, the production of lightweight, sustainable foams via eco-friendly processing routes necessitates an advanced understanding of the effects of reactive compatibilization on thermal and viscoelastic response of such foaming systems for enhancing their commercial application.

Several studies have been conducted on the melt-blending and foaming of PLA with secondary (bio)polymers, such as poly(ε-caprolactone) (PCL) [22], poly(hydroxybutyrate) (PHB) [23], polyhydroxybutyrate-valerate (PHBV) [24], poly(butylene succinate) (PBS) [25], poly(butylene adipate-co-terephthalate) (PBAT) [26], and poly(butylene succinate-co-adipate) (PBSA) [27]. Wu et al. [22] observed an increase in the crystallinity of compression-molded PLA-PCL blends upon incorporation of nanofillers. Abdelwahab et al. [23] reported improvement in crystalinity on compatibalized blends of PLA/PHB processed via compression-molding. Zhao et al. [24,28] prepared ScF-foamed physical blends of PLA/PHBV blends and composites, and studied their impact on crystallinity and thermomechanical properties. Yokohara and Yamaguchi [25] found that compression-molded PLA/PBS blends led to improved crystallinity and enhanced processability. Javadi et al. [26] studied the miscibility and the thermal and mechanical properties of ScF-foamed physical blends of PLA/PBAT and found improvements in damping ability. Ojijo et al. [27] studied compression-molded PLA/PBSA blends compatibilized via triphenyl phosphite (TPP) and observed a significant increase in crystallinity and thermal stability.

To the best of our knowledge, no studies have been undertaken on analyzing the effect of compatibilization and foaming on the crystallization and thermomechanical behavior of PLA/PBSA blends and composites processed via ScF injection molding. Hence, the objectives of this study are to understand the impact of compatibilization, ScF foaming, and the addition of talc on thermal behavior and viscoelastic properties. The (70:30) PLA/PBSA ratio was chosen as a model blend to study and understand the above-mentioned effects since it exhibited the highest crystallinity and improved properties, as detailed in Ojijo et al. [29].

## 2. Materials and Methods

Commercial polylactic acid (PLA) (3001 D) was purchased in pelletized form from Natureworks LLC (Minnetonka, MN, USA), with specific gravity of 1.24 and melt flow index of ~22 g/10 min. Commercially available poly (butylene succinate-co-adipate) (PBSA) (Bionolle #3001) pellets were sourced from Showa Denko (Tokyo, Japan), its specific gravity being 1.23 and melt flow index being 25 g/10 min. Talc used in this study (Mistrocell M90) was supplied by Imerys Talc (San Jose, CA, USA) with a mean diameter of 18.8 μm. Coupling agent triphenyl phosphite (TPP) was obtained from Sigma-Aldrich (Milwaukee, WI, USA), and 2 wt % TPP was used to compatibilize blends. Commercial-grade nitrogen was sourced from Airgas (Greenville, SC, USA) and used as a blowing agent in ScF-assisted injection molding.

### 2.1. Methods

A co-rotating twin screw extruder (ZSK 30 from Werner & Pfleiderer, Stuttgart, Baden-Württemberg, Germany) was used to compound the eight compositions prepared for this study, as listed in Table 1. Prior to extrusion, as-received PLA and PBSA pellets were dried at 75 °C for 8 h. Subsequently, talc and/or TPP were manually mixed with PLA/PBSA pellets in weight compositions listed in Table 1. Except for pure and talc-filled PBSA, extrusion for other compositions was subsequently carried out at temperature zones of 130/150/165/170/175 °C at a screw rotation speed of 35 rpm. Due to its low melting point (91 °C), extrusion of pure and talc-filled PBSA was carried out at temperatures of 100/125/135/140/145 °C.

Extruded pellets of all compositions were dried at 80 °C for 8 h prior to injection molding (IM). Conventional and ScF-assisted IM were carried out using an injection molding machine (Arburg Allrounder 3205, Lossburg, Baden-Württemberg, Germany), which was equipped with a Trexel Series II ScF dosing system, Wilmington, MA, USA. Injection molding parameters are listed in Table 2, while IM melt temperatures were reduced for pure PBSA to 100/140/145/135/125 °C—similar to extrusion due to its low melting point. However, in the metering zone, temperatures had to be increased to ensure a consistent pressure drop during gas dosage. Weight % of supercritical N_2_ was calculated by Equation (1):

(1) $$\mathrm{wt}\;\%\;\mathrm{ScF}=\;\frac{\dot{\dot{m}tX\left(27.8\right)}}{m}$$

where $\dot{m}$ is the mass flow rate of ScF (kg/h), *t* is the ScF dosage time (s), *m* is the shot weight (g), and 27.8 is a conversion factor.

A total of 24 samples (solid IM and ScF IM with two gas dosages, 0.73 and 0.94 wt %)—3 per each composition listed in Table 1—were prepared. Subsequently, injection-molded specimens were labeled as “*XX*-*Y*”, where *XX* corresponds to nomenclature mentioned in Table 1, while “*Y*” indicates the nature of the sample as solid or foamed, with “*S*” referring to the solid injection-molded sample, “1” referring to the ScF-assisted injection-molded sample obtained at a ScF gas dosage of 0.73 wt %, and “2” referring to the ScF-assisted injection-molded sample at a gas dosage of 0.94 wt % ScF.

The IM samples were characterized using gel permeation chromatography (GPC), differential scanning calorimetry (DSC) and a dynamic mechanical analyzer (DMA) in order to understand the effect of physical and chemical compatibilization, the addition of fillers, and the ScF foaming of PLA and PBSA on thermal and viscoelastic properties.

### 2.2. Gel Permeation Chromatography

Number-average molecular weight (*M*_n_) and polydispersity index (PDI) for solid injection-molded samples were determined via gel permeation chromatography (GPC) on Waters GPC equipped with a UV–Vis and RI detector. Chloroform was used as an effluent (flow rate of 1.0 mL/min) at 33 °C. All samples were prepared as 0.5% (*w*/*v*) solutions in chloroform, with ~50 µL of sample injected into the GPC. Prior to injection, the dissolved solution was filtered using a 0.2 μm PTFE filter. Calibration was done using narrow molecular weight polystyrene standards ranging from ~436 to ~990,500 Daltons.

### 2.3. Differential Scanning Calorimetry

A differential scanning calorimeter (TA Instruments, Q2000, New Castle, DE, USA) was used to study the crystallization behavior of all 24 samples. About 7–9 mg of sample was taken in hermetically sealed aluminum pans. Samples were subjected to heating/cooling/heating cycles at 5 °C/min, beginning with heating from −100 to 200 °C (to remove any thermal history from processing), held isothermally for 5 min, cooled to −100 °C, and subsequently heated to 200 °C. The temperature of cold crystallization (*T*_cc_), the melting temperature (*T*_m_), the apparent melting enthalpy ($∆H_{m}$), and the enthalpy of cold crystallization ($∆H_{\mathrm{cc}}$) were determined via DSC curves. The crystallinity of PLA and PBSA were calculated by Equation (2):

(2) $$\chi_{C}\left(\%\;\mathrm{crystallinity}\right)=\frac{∆H_{m}-∆H_{\mathrm{cc}}}{∆H^{0}}\;\times \;\frac{100}{W}$$

where $∆H_{m}$(PLA) and $∆H_{m}$(PBSA) are the enthalpies of melting per gram of 100% crystal (perfect crystal) of PLA and PBSA (93.7 and 142 J/g), respectively, and W is the weight fraction of either PLA or PBSA in the blend [30,31].

### 2.4. Dynamic Mechanical Analyzer

Dynamic mechanical analysis was carried out using TA Q800 Dynamic Mechanical Analyzer, New Castle, DE, USA. Rectangular specimen (4 mm × 8 mm × 70 mm) were cut from the gauge length of injection-molded specimen and tested in dual cantilever mode. Samples were tested at temperatures between −50 and 100 °C at a heating rate of 3 °C/min at a 1 Hz frequency and a 0.1% strain amplitude in order to determine glass transition temperature, storage, and loss moduli.

## 3. Results

### 3.1. Gel Permeation Chromatography

Number-average molecular weight (*M*_n_), weight-average molecular weight (*M*_W_), and polydispersity index (PDI) were determined for all solid samples and tabulated in Table 3. As can be seen,

(a) *M*_n_ of PLA (A-S) and PBSA (B-S) were obtained as ~90,000 and ~62,000 Daltons, respectively;
(b) While the *M*_n_ of the physical blends (P-S) (~64,000 Daltons) was found to be between that of PLA (A-S) and PBSA (B-S), compatibilized blends (C-S) showed a higher *M*_n_ (~101,796 Daltons) compared to both P-S (by over ~40,000 Daltons) and A-S (by over ~10,000 Daltons);
(c) Addition of talc resulted in a marginal reduction in the *M*_n_ of PLA (AT-S) and a marginal increase in the *M*_n_ of all other compositions—namely, BT-S, PT-S, and CT-S—compared to its non-talc counterparts—within talc-filled compositions, the *M*_n_ of PT-S (~79,026 Daltons) was found to be between those of AT-S (~85,083 Daltons) and BT-S (~66,173 Daltons), while CT-S (~108,483 Daltons) showed an improvement over all three compositions;
(d) PDI for pure and talc-filled compatibalized blends (C-S and CT-S) was found to be narrower than that for other compositions.

### 3.2. Differential Scanning Calorimetry

#### 3.2.1. First Heating Thermograms

Temperature of cold crystallization (*T*_cc_), melting temperature (*T*_m_), and their respective ethalpies of cold crystalization ($∆H_{\mathrm{cc}}$) and ($∆H_{m}$)—as obtained from first heating thermograms—are reported for pure polymer (Table 4) and for polymer blends (Table 5), respectively.

Among individual polymer compositions, pure PLA compositions showed *T*_cc_ values of ~97.3 °C (A-S), ~99.6 °C (A-1), and ~100 °C (A-2), respectively. Compared to the physical blends (*T*_cc_ ~ 81 °C), chemically compatibilized blends exhibited a significant reduction in *T*_cc_ (*T*_cc_ ~ 71 °C). The introduction of talc in the PLA samples led to a reduction in *T*_cc_ compared to pure PLA compositions, such as from ~97.3 °C (A-S) to ~90.2 °C (AT-S) or from ~100 °C (A-2) to ~91.4 °C (AT-2), respectively. In the case of physical and chemically compatibilized PLA–PBSA blends, the addition of talc did not significantly alter *T*_cc_ compared to non-talc counterparts. All *T*_cc_ values observed in the blended samples correspond to the PLA component, while PBSA samples (both talc-filled and non-talc) did not exhibit any *T*_cc_ value.

With regard to melting behavior, while all PLA samples showed a single melting peak at ~168 °C, all PBSA samples showed a single melting peak at ~92 °C. However, in the case of blended samples, two melting peaks were observed, one each corresponding to melting temperatures of PBSA and PLA, respectively. While physically blended samples showed melting peaks at ~92 and ~167 °C, chemically blended samples showed a shift in both melting peaks to ~88 and ~155 °C. The addition of talc and/or ScF was not found to result in any significant shift in melting point (*T*_m_).

With respect to crystallinity, solid PLA (A-S) exhibited a crystallinity of ~20.19%, while its foamed counterpart (A-2) showed a higher crystallinity of ~27%. A similar increase in crystallinity of PBSA was observed from ~33% (B-S) to ~35% (B-1 and B-2). While physically blended foamed samples (P-1, P-2, PT-1, and PT-2) showed a crystallinity of ~15%–18%, their chemically conjugated foamed counterparts (C-1, C-2, CT-1, and CT-2) exhibited crystallinity levels of ~20%–28%. The addition of talc was observed to improve crystallinity to varying degrees for all compositions compared to their non-talc counterparts, both for solid and foamed compositions. For example, while AT-S showed improvement in crystallinity by ~5% compared to A-S, physical blends showed improvement by ~2% compared to their non-talc counterparts.

#### 3.2.2. Second Heating Thermograms

**Figure 1:**
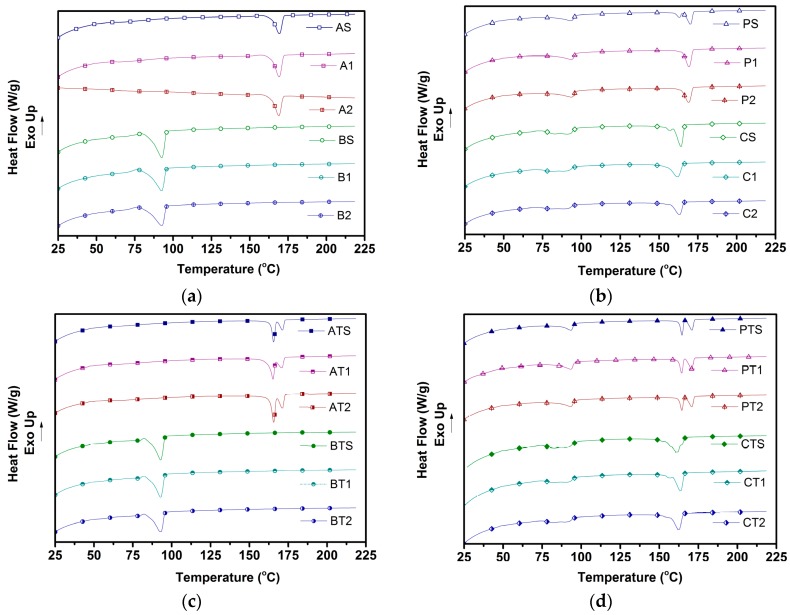
Second heating Differential Scanning Calorimetry (DSC) curves of (**a**) non-talc pure; (**b**) non-talc blend; (**c**) talc pure; and (**d**) talc blend compositions.

The second heating thermograms for all samples is shown in Figure 1a–d, while the glass transition temperature (*T*_g_), the melting temperature (*T*_m_), and crystallinity levels (%) obtained from these thermograms are reported for the pure polymers (Table 6) and for the blends (Table 7).

*T*_g_ for solid PLA (A-S) was observed to be 63.8 °C with marginal decrease for both foamed compositions (A-1 and A-2). A similar trend was observed for PBSA, with *T*_g_ gradually reducing from −41.8 °C (solid PBSA or B-S) to lower values for both foamed counterparts (B-1 and B-2). However, *T*_g_ for both physical and chemical blends could not be observed at the ramp rate tested in this study.

Melting point (*T*_m_) was observed to be 169 °C for all pure PLA compositions (A-S, A-1, and A-2), albeit with the addition of talc (AT-S, AT-1, and AT-2) resulting in obtainment of bimodal melting peaks at ~165 and ~171 °C, respectively. However, PBSA showed a consistent single melting peak of ~92 °C for all PBSA samples (both talc-filled and non-talc). Physically blended solid samples showed three melting peaks: one at ~94.6 °C (corresponding to PBSA), and bimodal peaks at ~165 and ~170 °C (corresponding to PLA). Chemically compatibilized solid samples exhibited a similar trend, with melting peaks at ~90.0 °C (corresponding to PBSA), and bimodal peaks at ~158.8 and ~164.1 °C (corresponding to PLA). Interestingly, their foamed counterparts (P-1, P-2, C-1, and C-2) showed only two peaks at ~93 °C (corresponding to PBSA) and ~169 °C (a single peak corresponding to PLA). The addition of talc to blends resulted in the obtainment of bimodal peaks (corresponding to PLA) in physical blends (PT-S, PT-1, and PT-2), in stark contrast to a single melting peak (corresponding to PLA) in chemical blends (CT-S, CT-1, and CT-2).

With regard to crystallinity, the addition of talc led to an increase in crystallinity of PLA and PBSA samples by ~4% and ~5%, respectively. The crystallinity of the PLA component in the blends was observed to enhance by ~4% for both physical and chemically compatibilized blends, with the effect of talc being more pronounced for compatibilized blends. Chemically compatibilized foamed blends (C-1, C-2, CT-1, and CT-2) showed higher crystallinity vs. their physically foamed counterparts (P-1, P-2, PT-1, and PT-2, respectively). However, the enhancement in crystallinity due to the use of ScF foaming was not as pronounced as that due to the addition of talc, with ~50% crystallinity observed for talc-filled chemically compatibilized samples (CT-S, CT-1, and CT-2).

### 3.3. Dynamic Mechanical Analysis

**Figure 2:**
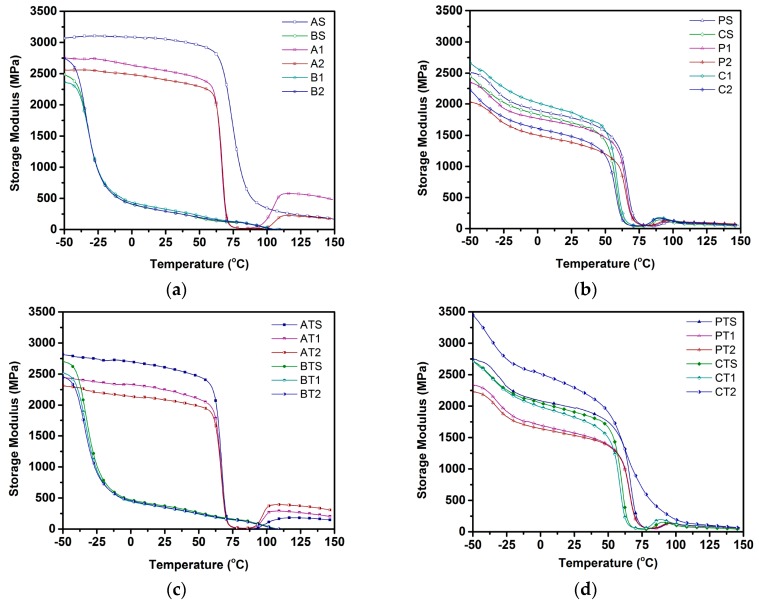
Storage modulus of (**a**) non-talc pure; (**b**) non-talc blend; (**c**) talc pure; and (**d**) talc blend compositions.

**Figure 3:**
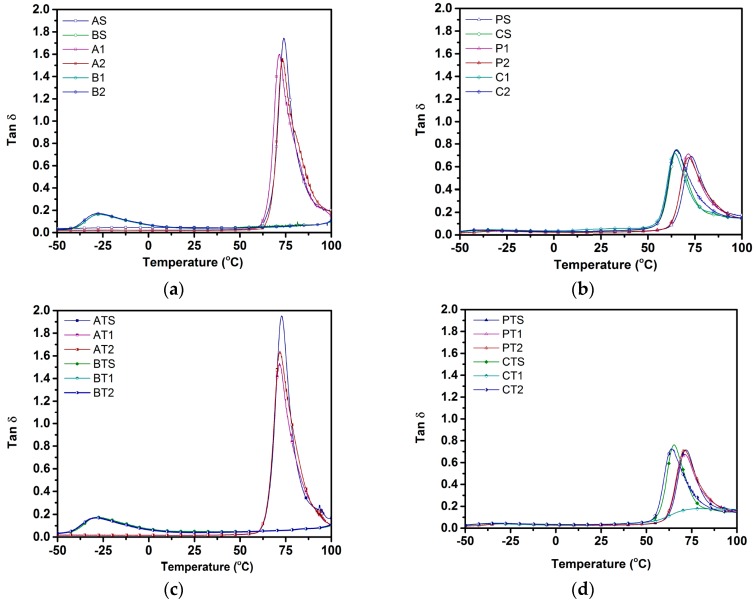
Tanδ of (**a**) non-talc pure; (**b**) non-talc blend; (**c**) talc pure; and (**d**) talc blend, compositions.

Viscoelastic behavior of all samples was studied using DMA to track temperature dependence of storage modulus and tanδ. Figure 2a–d show storage modulus curves as a function of temperature, while Figure 3a–d show dependence of tanδ on temperature. All samples exhibited a decline in storage modulus with an increase in temperature. While a plateau region was observed for all PLA samples up to its *T*_g_ of ~63 °C, similar plateau regions were not observed for the blends and PBSA samples. Solid and foamed physical blends (P-S, P-1, and P-2) exhibited glass transition at ~62 °C (corresponding to PLA), while compatibilized blends (C-S, C-1, and C-2) exhibited a shift in *T*_g_ to ~53 °C (corresponding to PLA). *T*_g_ values for all PBSA samples (B-S, B-1, B-2, BT-S, BT-1, and BT-2) was the same as that of PBSA at ~−40 °C.

The storage moduli at −50 and 25 °C for all compositions is reported in Table 8. The storage modulus at −50 °C was observed to reduce upon the use of ScF for both talc-filled and non-talc PLA and PBSA samples, with non-talc-based PLA compositions showing a higher storage modulus vs. non-talc-filled PBSA or blend compositions (Figure 2a,b). Figure 2c,d shows that, in sum, talc-filled samples (excluding those of PLA) exhibited a higher storage modulus compared to their non-talc counterparts. While solid physical and chemically compatibilized blends exhibited distinct storage moduli of ~2500 MPa at −50 °C, microcellular physical blends showed lower storage moduli (2315 and 2028 MPa), while chemically foamed blends showed higher storage moduli. Among foamed compatibilized blends, non-talc blends at a lower ScF gas dosage (C-1) exhibited the highest storage modulus among all non-talc blends, while CT-2 showed the highest storage modulus among all 24 samples.

The post-glass transition hump observed in Figure 2a–d is analogous to a cold crystallization temperature (*T*_cc_) [32]. While solid PLA samples did not show any *T*_cc_, the foamed PLA samples (A-1 and A-2) exhibited a *T*_cc_ of ~108 and ~109 °C, respectively. In contrast, no PBSA sample showed any *T*_cc_. With regard to the non-talc-filled blend samples, the physically blended samples showed a *T*_cc_ at ~96 °C, while chemically compatibilized blends exhibited a lower *T*_cc_ at ~89 °C. The addition of talc was observed to lead to a reduction in *T*_cc_ for the PLA samples (AT-1 and AT-2) to ~101 and ~102 °C, respectively, with a reduction in *T*_cc_ for the physically blended samples (~95 °C) and chemically compatibilized samples (~85 °C). Interestingly, CT-2 did not show any cold crystallization temperature.

Tanδ is the ratio of loss modulus to storage modulus. Table 9 tabulates the glass transition temperature (*T*_g_) corresponding to tanδ peaks—as this is often analogous to *T*_g_ of the polymer—and the area under the tanδ curve. As shown in Figure 3a, the *T*_g_ of the PLA is ~75 °C; however, with chemical compatibilization, it was observed to undergo a significant shift to lower temperatures (~65 °C) (Figure 3b). While the blend compositions showed no *T*_g_ corresponding to PBSA, the physical blends exhibited a *T*_g_ of ~72 °C, and compatibilized blends exhibited a relatively low *T*_g_ (~64 °C), both corresponding to the *T*_g_ of the PLA. The addition of talc was not found to result in any significant shift in *T*_g_ of any composition based on their tanδ peaks, while the area under the tanδ curve was observed to reduce for both physical and chemically compatibilized blends compared to the pure PLA-based compositions.

## 4. Discussion

### 4.1. Compatibilization Mechanism

Most physical blends of PLA with toughened secondary polymers (including PBSA) are thermodynamically immiscble [29]. It is common practice to add compatibilizers in order to improve the compatibility of these immiscible blends. An addition of compatibilizer results in a reduction of interfacial tension due to the formation of either a block or graft copolymer at interfaces within the blend, depending on the kind of compatibilizer used [13,33]. For example, an addition of compatibilizers possessing reactive end groups will result in the formation of block copolymers (with a substantial increase in *M*_n_) [34], while an addition of compatibilizers with reactive pendant groups (such as TPP) will generally result in the formation of graft/branched copolymers [35].

**Figure 4:**
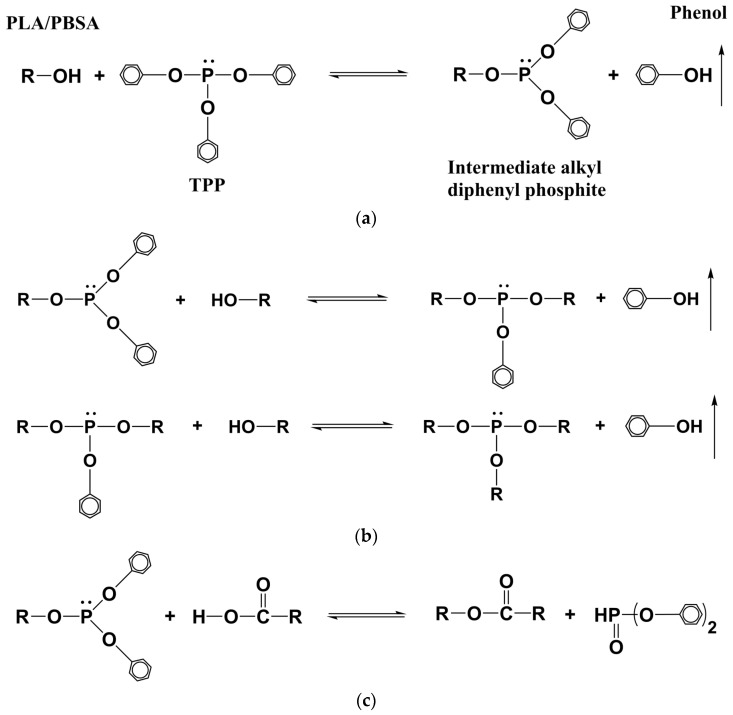
Reaction schemes of (**a**) the initiation of the reaction between triphenyl phosphite (TPP) and polylactic acid (PLA)/poly(butylene succinate-co-adipate) (PBSA); (**b)** the propagation reaction inducing a possible branching mechanism between the hydroxyl ends of PLA/PBSA polymeric chains; and (**c**) the propagation reaction inducing a possible chain extension mechanism between the hydroxyl chain ends of PLA/PBSA polymeric chains. Adapted with permission from [27]. Copyright 2013, American Chemical Society.

Different researchers have undertaken studies on the effect of compatibilizers with reactive pendant groups (such as TPP) on polyester-based systems and have proposed two reaction mechanisms—one by Jacues et al. [35] and the other by Aharoni et al. [36]. These reaction mechanisms have a strong impact on the compatibilization of polymer blends and their properties. Hence, any understanding of how the addition of TPP influences the compatibilization of PLA and PBSA in this study needs to be taken into account. In both of the above-mentioned reaction mechanisms, the first step is the preferential reaction of hydroxyl end-groups of PLA/PBSA with TPP via the displacement of one of TPP’s phenoxy groups, as shown in Figure 4a. This leads to the formation of an intermediate phosphorus-containing compound (intermediate alkyl diphenyl phosphite). The second step can be either of the two reaction mechanisms depicted in Figure 4b,c.

In the first reaction mechanism, the second step involves a multi-substitution reaction of intermediate alkyl diphenyl phosphite whereby phenoxy groups are replaced with alkyl groups along with the elimination of phenol, as shown in Figure 4b. It is highly likely that this reaction continues until phosphorus serves as a binding point for the occurrence of grafting/branching [33]. In contrast, the second mechanism involves ester linkages from polymers, with phenoxy groups of intermediate product reacting with carboxyl groups of PLA/PBSA (instead of hydroxyl end-groups), leading to a chain extension without P atoms becoming part of the polymeric chain (Figure 1c).

In all of the above-mentioned reaction schemes, chain extension and/or branching may occur. In our case, compatibilized blends show a marginal increase in molecular weight (Table 3) compared to PLA, indicating that branching is a major reaction pathway. This has been observed in a previous study conducted by Jacues et al. [35], where 2 wt % TPP was used to melt-blend PET/PBT in a ratio of 70:30 [35]. The authors observed a small increase in *M*_n_, accompanied by branching of both polyesters, as proven by an increase in torque oscillations. Harada et al. [37] observed a similar trend for compatibilized PLA–PBSA blends involving the use of lyscine triisocyanate as a coupling agent, with cross-linking behavior being reported and accompanied by a small increase in the *M*_n_ of the PLA blends. Further studies involving multi-detector gel permeation chromatography GPC using viscometry and light scattering might be required to ascertain the exact nature of branching.

### 4.2. Crystallization & Melting Behavior

Typically, semi-crystalline polymers such as PLA and PBSA can exhibit three kinds of crystallization behaviors—melt crystallization, cold crystallization, and recrystallization—depending on the heating/cooling rate adopted. Melt crystallization refers to the formation of crystals during cooling. Cold crystallization is the ability of amorphous domains to crystallize during heating, while re-crystallization refers to the reorientation of crystals formed during melt/cold crystallization [6,38]. In our study, the observation during the first heating pertains to the behavior of injection-molded samples, which were typically subjected to high cooling rates (~200 °C/min), leading to insufficient time available for crystallization. The second heating cycle erases the prior thermal history of the samples while subjecting them to a low cooling rate in the first cooling cycle (5 °C/min), and is indicative of the behavior of the nascent material [14]. Hence, the differences observed in the behavior of all samples between both heating cycles in this study, such as (1) the occurrence of cold crystallization only in the first heating cycle; (2) the presence of a single melting peak in the first cycle vs. double melting peaks in the second heating cycle (both corresponding to PLA) in few samples; and (3) an enhanced crystallinity of samples after the second heating cycle; all of which can be attributed to the stark difference in cooling rate.

The presence of *T*_cc_ (corresponding to PLA) reported in Table 4 and Table 5 during the first heating cycle and its absence in the second heating cycle was because all amorphous molecular domains had crystallized in the first cooling cycle upon use of a slow cooling rate (5 °C/min). This is in good agreement with the observed increase in the crystallinity of blends from the first heating cycle to the second one, as it indicates that amorphous domains crystallized during the first cooling cycle. Pilla et al. [14] observed similar behavior in the case of PLA/MWCNT (multi-wall carbon nanotubes) composites.

The absence of cold crystallization (corresponding to PBSA) in PBSA samples and blends could be due to several factors. First, PBSA molecules tend to undergo a faster rate of crystallization during cooling, leading to an absence of amorphous domains that could crystallize during reheating [39]. Second, in the case of blend samples, the presence of stiff PLA chains hinders the cold crystallization of PBSA [29], further making its occurrence impossible in blends. With regard to blends, the physical PLA/PBSA blends showed a reduction in *T*_cc_ compared to the pure PLA samples, which could be attributed to the possible intermingling of chains of both polymers at the interfaces, resulting in the early onset of crystallization [29]. A further decrease in *T*_cc_ was observed for chemically compatibilized blends to ~71 °C, which could be attributed to the enhanced compatibility between PLA and PBSA chains [27].

The reduction in melting temperatures in the compatibilized blend of around ~7 °C in both heating cycles was due to a stronger interaction between PLA and PBSA chain segments upon the addition of triphenyl phosphite (TPP), as TPP enhances the mobility of PLA chain segments [27]. This finding is in good agreement with Ojijo et al. [27], who observed a similar lowering in the *T*_m_ (to ~152 °C) of compatibilized PLA/PBSA blends prepared via use of similar coupling agents.

Furthermore, solid blends (P-S and C-S) exhibited double melting peaks that were due to the melting of PLA crystals with different morphologies [15]. Ojijo et al. [29] had observed that PBSA in molten form has a nucleating effect on the crystallization of PLA, forming crystals of different sizes and morphologies. Hence, the observed double melting peaks was mainly due to the nucleating effect of PBSA. This is due to the inability of simultaneous crystallization of both polymers occurring due to the large difference in their melting temperatures. However, their foamed counterparts (P-1, P-2, C-1, and C-2) showed only one melting peak corresponding to a melting of PLA. This indicates that foaming had a strong impact on the reorientation of crystal structures, leading to the formation of highly ordered crystals, even as TPP induced strong compatibilization between PLA and PBSA.

The addition of talc also resulted in the obtainment of double melting peaks in PLA (AT-S, AT-1, and AT-2) and physical blends (PT-S, PT-1, and PT-2), which could be due to the heterogeneous nucleation effect of talc particles resulting in the obtainment of varying crystal sizes, which is in agreement with other literature [40,41,42]. Interestingly, compatibilized blends showed only one melting peak upon the addition of talc—in stark contrast with the above-mentioned observation. This can be explained by the reinforcing effect of talc, which enhances bulk crystallinity without impacting crystal size, as observed by Tanniru and Misra et al. [43] for CaCO_3_-reinforced PE composites.

The crystallinity of foamed compatibilized blends was higher compared to the physically blended counterparts, a phenomenon also observed by Yang et al. [44] on PLA–PBSA compatibilized blends, who attributed this to branching sites acting as nucleation points, leading to a higher probability of nucleation compared to the physical blends. This is in good agreement with our molecular weight results, measured by GPC, indicating a possible occurrence of grafting/branching. For both physical and chemically compatibilized blends, foaming resulted in a higher degree of crystallinity. This could also be attributed to the biaxial extensional flow of ScF affecting the orientation of polymer molecules around cell walls due to foaming, leading to strain-induced crystallization, which results in an increase in the final crystallinity, as observed by Ameli et al. [45]. A similar trend was also observed by Zhai et al. [46] in using chemical foaming agents to foam polycaprolactone. The addition of talc led to an increase in crystallinity for most samples, which could be attributed to the nucleating effect of talc.

### 4.3. Viscoelastic Behavior

The storage modulus is a measure of energy storage and recovery exhibited during cyclic deformation, reflecting the elastic moduli of a material. In general, the storage modulus of any given material can be altered via addition of fillers. Generally, an addition of inorganic fillers is known to enhance the storage modulus of PLA [41,42,47]. However, the opposite trend was observed in the pure PLA in this study (A-S and AT-S), which could be due to the inability of talc to exhibit a reinforcing effect. In general, the reinforcing effect of talc is more pronounced in a material exhibiting less stiffness, as explained by Tanniru and Misra [43], who have observed a similar effect of fillers on polymeric materials with reduced stiffness. The pure PLA used in this study exhibited a storage modulus of 3050 MPa at 40 °C, which is far higher than the storage modulus of both pure PLA (2450 MPa) and PLA containing 10 wt % of silane-treated wood fiber (2556 MPa) reported by Pilla et al. [39]. This excessively high storage modulus of pure PLA used in our study might be a contributing factor towards the lack of any reinforcing effect of talc in the talc-filled PLA samples. However, the opposite trend was observed for both PBSA-based and blended samples due to the elastomeric nature and resultant lower stiffness of PBSA, resulting in an improvement in the storage modulus upon the addition of talc. Among solid blends, compatibilized blends showed a lower storage modulus vis-à-vis physical blends, primarily due to the hindrance in chain movement on account of the possible branching that prevented chain realignment/packing, as observed by Khonakdar et al. in crosslinked HDPE (High-density polyethylene) [48]. Similar phenomena was observed by Ibrahim et al. [49] for cross-linked PLA/PCL (poly(ε-caprolactone)) blends compared to physical PLA/PCL blends, and was attributed to the creation of voids in the system upon the formation of the crosslinking network. The compatibilized foamed blends showed a higher storage modulus in this study compared to their physically foamed counterparts, which could be attributed to the higher crystallinity (observed in Table 5 and Table 7) due to the synergistic effect of TPP and ScF on crystallinity.

With regard to glass transition temperature, the absence of the plateau region in the storage modulus curve was observed for blend compositions, and can be attributed to the extremely low *T*_g_ value of PBSA (~−40 °C). Similar observations have been made in another study by Ibrahim et al. [49], where no plateau region was observed in the storage modulus curve of PLA/PCL blends on account of a low *T*_g_ value of PCL (~−60 °C). Ojijo et al. [29], in their study on PLA/PBSA blends, also observed similar trends, and attributed the absence of a plateau region to an increased mobility of PBSA chains above its *T*_g_ (~−40 °C), leading to a lowering of blend stiffness.

Storage modulus was observed to undergo a sudden increase after a glass transition, corresponding to PLA, for all PLA-containing compositions. This increase was analogous to the cold crystallization from the first heating cycle of DSC, which is in accordance with Zhang et al. [32] where cold crystallization was observed for both individual and blend compositions (PLA–PHBV (polyhydroxybutyrate-valerate)–PBS (poly(butylene succinate))) after glass transition. The appearance of T*_cc_* can be explained by the fact that, for both individual and blend samples, the DMA (Dynamic Mechanical Analyzer) tests were undertaken on injection-molded samples that possessed low crystallinity levels due to the use of high cooling rates (as explained in Section 4.2). Such low crystallinity levels indicated a significant presence of amorphous domains available for crystallization during heating in DMA, allowing them to crystallize post-glass transition, along with an associated sudden increase in storage moduli. With regard to blend compositions, the presence of molten PBSA as nucleating agents acted as an additional factor in enhancing the crystallinity and the subsequent jump in storage moduli [29].

The trends observed for the glass transition temperature (*T*_g_) in storage moduli curves and tanδ curves were in good agreement with each other for all samples (Figure 3 and Figure 4). In the tanδ curve, a peak was observed in the region where, with increases in temperature, the rate of the decrease in storage modulus was higher than that of the loss modulus. Temperatures corresponding to the tanδ peak is often considered as *T*_g_. Interestingly, *T*_g_ was not observed for the PBSA component in all blend samples due to the locking of PBSA chains by hard PLA segments, thus preventing their motion. Additionally, the use of a lower weight fraction of PBSA meant that a higher share of PBSA chains were restricted by PLA chain segments, ensuring that no *T*_g_ corresponding to PBSA was observed for blend compositions [29].

Glass transition temperature of blend samples gives us insight into the miscibility of pure polymers constituting the blends. *T*_g_ is typically dependent on the polymer composition of blends, and lies between the *T*_g_ values of pure constituents for a completely miscible blend [50]. To obtain clarity on the miscibility and effect of TPP on PLA–PBSA blends, a simplified version of the Gordon–Taylor (G-T) equation (Equation (3)) [51] was applied to *T*_g_ obtained from tanδ.

(3) $$T_{g}=\frac{W_{1}T_{g1}+kW_{2}T_{g2}}{W_{1}+kW_{2}}\;$$

**Figure 5:**
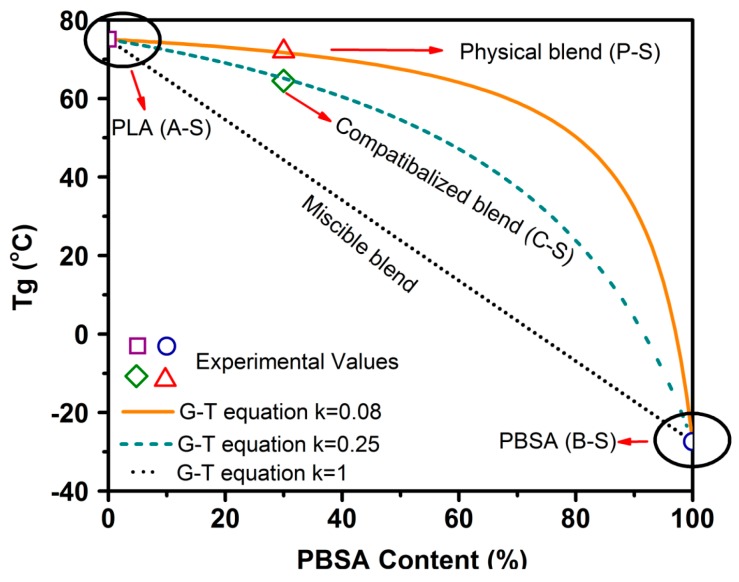
Comparison of the experimental and theoretical *T*_g_ values of PLA–PBSA blends.

Here, $T_{g1}$ and $T_{g2}$ are the glass transition temperatures of pure components PLA and PBSA, respectively, while $W_{1}$ and $W_{2}$ are the wt % of PLA and PBSA, respectively, and k is a curve-fitting factor representing the miscibility of the system, with k = 1 indicating the complete miscibility of the polymers and the lower/higher values of *k* indicating poor miscibility. Figure 5 depicts $T_{g}$ of different blend compositions. Observed $T_{g}\;$values for A-S, P-S, and B-S (~75.1, ~72, and ~−27.4 °C) and A-S, C-S, and B-S (~75.1, ~64, and ~−27.4 °C) were plotted as the *T*_g_ of the talc-filled and ScF-foamed blends, all of which were found to overlap (Table 8). These observed values were closer to the G-T curve for k = 0.08 and k = 0.25, where the curve-fitting parameter k showed a value of 0.08 for the physical blends, indicating the poor miscibility of PLA and PBSA, as they are thermodynamically immiscible [52]. However, an addition of 2 wt % TPP shifted the $T_{g}$ of PLA–PBSA blends to around 64 °C, with the k value of 0.25 used to curve fit the G-T equation; this higher value of k indicates the possibility of enhanced compatibilization.

## 5. Conclusions

Compatibilized blends of PLA and PBSA were successfully processed using TPP via reactive extrusion and foamed via ScF-assisted injection molding technology. The compatibilization was verified via an improvement in *M*_n_ using GPC, a shift in $T_{g}$ using DSC, and an improved miscibility as shown by the G-T equation. Thermal properties of solid and foamed samples, studied using DSC, revealed that the addition of talc/compatibilizer and the use of ScF foaming had a significant impact on crystallinity, melt, cold crystallization, and glass transition temperatures. Compatibilized ScF-foamed blends showed an improvement in crystallinity by ~10% over their physical blend unfoamed counterparts. The viscoelastic properties of the samples revealed further evidence of compatibilization, as verified by the G-T equation. Furthermore, compatibilized foamed blends showed superior storage moduli compared to their physically foamed counterparts due to the synergistic effect of TPP and ScF on crystallinity.

## Figures and Tables

**Table 1:** Design of experiment (DOE) formulations in this study (ratio) ^1^.

Sample	Nomenclature	PLA	PBSA	Talc
Pure PLA	A	100	-	-
Pure PBSA	B	-	100	-
Physical Blend	P	70	30	-
TPP Compatibilized blend	C	70	30	-
PLA + Talc	AT	95	-	5
PBSA + Talc	BT	-	95	5
Physical Blend + Talc	PT	70	30	5
TPP Compatibilized blend +Talc	CT	70	30	5

^1^ Polylactic acid (PLA), poly(butylene succinate-co-adipate) (PBSA), triphenyl phosphite (TPP).

**Table 2:** Experimental conditions for solid and supercritical fluid assisted injection molding (ScF IM).

Parameter	Solid Molding	Foamed Molding
Back pressure (MPa)	10	80
Melt temperatures (°C)	155/165/175/185/195	155/165/175/185/195
Injection pressure (bar)	2500	2500
Injection speed (cm^3^/s)	65	65
Holding pressure (bar)	800	0
Holding time (s)	3	0
Cooling time (s)	60	60
Gas dosage (wt %)	0	0.73 and 0.94

**Table 3:** *M*_n_, *M*_W_, polydispersity index (PDI), and area for all compositions.

Sample	*M*_n_ (Daltons)	PDI
A-S	90,039	1.8
B-S	62,175	2.1
P-S	64,685	2.1
C-S	101,796	1.4
AT-S	85,083	1.7
BT-S	66,173	2.0
PT-S	79,026	1.8
CT-S	108,483	1.4

**Table 4:** The thermal behavior of injection-molded pure samples obtained from 1st heating thermograms.

Sample	*T*_cc_ (°C)	Δ*H*_cc_ (J/g)	*T*_m_ (°C)	Δ*H*_m_ (J/g)	% Crystallinity
A-S	97.3	26.42	168.8	45.34	20.19
A-1	99.6	26	168.7	46	21.34
A-2	100.8	22.41	168.7	47.76	27.05
AT-S	90.2	19.23	168.4	43.51	25.91
AT-1	90.9	17.63	168.2	43.04	27.11
AT-2	91.4	18.19	168.2	44.22	27.78
B-S	-	-	92.9	47.22	33.25
B-1	-	-	92.9	49.99	35.20
B-2	-	-	92.9	50.75	35.73
BT-S	-	-	93.0	49	34.50
BT-1	-	-	92.8	51	35.91
BT-2	-	-	92.9	52	36.61

**Table 5:** Thermal behavior of injection-molded blends obtained from 1st heating thermograms.

Sample	$T_{cc}$ (°C)	Δ*H*_cc_ (J/g)	$T_{m1}^{PBSA}$ (°C)	$T_{m2}^{PLA}$ (°C)	Δ*H*_m_ (J/g)	% Crystallinity
P-S	81.2	21.97	92.8	167.8	32.14	15.50
P-1	81.1	21.07	92.1	167.8	32.03	16.70
P-2	81.0	20.97	92.5	167.9	31.62	16.23
PT-S	81.1	18.5	92.8	167.8	29.85	17.30
PT-1	81.0	16.29	93.0	167.8	28.23	18.20
PT-2	80.9	16.48	93.1	167.5	29.19	19.37
C-S	71.3	25.33	88.0	155.6	35.83	16.00
C-1	71.4	20.868	88.6	158.0	33.29	18.93
C-2	71.0	18.04	88.1	155.5	34.07	24.40
CT-S	72.1	22	89.0	156.8	33	16.77
CT-1	73.8	14.27	88.7	154.8	33.18	28.83
CT-2	74.2	16.7	89.0	155.2	34.27	26.78

**Table 6:** Thermal properties of injection-molded samples obtained in 2nd heating thermograms.

Sample	*T*_g_ (°C)	$T_{m}^{PBSA}$ (°C)	$T_{m}^{PLA}$		Δ*H*_m_ of PBSA (J/g)	Δ*H*_m_ of PLA (J/g)	% Crystallinity PBSA	% Crystallinity PLA
			*T*_m1_ (°)	*T*_m2_ (°C)				
A-S	63.8	-	169.4	-	-	36.69	-	39.15
A-1	63.1	-	169.1	-	-	39.17	-	41.8
A-2	61.9	-	168.8	-	-	38.78	-	41.3
AT-S	63.2	-	165.6	171.5	-	40.84	-	43.58
AT-1	63.1	-	165.1	170.6	-	43.01	-	46.90
AT-2	62.7	-	165.6	171.2	-	44.35	-	47.33
B-S	−41.8	92.7	-	-	37.24	-	26.22	-
B-1	−42.8	92.4	-	-	43.16	-	30.39	-
B-2	−42.1	92.7	-	-	44.37	-	31.24	-
BT-S	−42.3	-	-	-	45.4	-	31.97	-
BT-1	−43.4	-	-	-	53.06	-	37.36	-
BT-2	−43.0	-	-	-	54.29	-	38.23	-

**Table 7:** Thermal properties of injection-molded samples obtained in 2nd heating thermograms.

Sample	$T_{m}^{PBSA}$ (°C)	$T_{m}^{PLA}$		Δ*H*_m_ of PBSA (J/g)	% Crystallinity PBSA	Δ*H*_m_ of PLA (J/g)	% Crystallinity PLA
		*T*_m1_ (°C)	*T*_m2_ (°C)				
P-S	94.65	165.6	170.10	15.46	36.6	28.53	43.49
P-1	93.54	169.33	-	12.66	23.80	25.11	38.28
P-2	93.63	169.23	-	13.45	24.47	25.21	38.43
PT-S	93.45	164.48	170.62	13.17	30.91	27.74	40.29
PT-1	93.25	164.20	170.06	13.76	32.30	27.90	42.53
PT-2	93.29	164.40	171.06	14.64	34.36	26.83	42.90
C-S	90.03	158.81	164.14	11.13	34.51	34.51	42.90
C-1	87.75	160.39	-	9.39	22.042	30.39	46.33
C-2	88.61	160.97	-	10.67	25.04	30.74	46.86
CT-S	90.67	160.23	-	9.916	23.27	35.76	54.52
CT-1	90.96	163.54	-	9.961	23.38	34.86	53.14
CT-2	90.28	162.71	-	9.54	22.39	34.49	52.58

**Table 8:** Storage moduli of all compositions at −50 and 25 °C.

Sample	Storage Modulus (MPa) at −50 °C			Storage Modulus (MPa) at 25 °C		
	Solid	ScF 1	ScF 2	Solid	ScF 1	ScF 2
A	3069	2731	2555	3063	2552	2403
B	2500	2365	2650	292	336	295
P	2510	2315	2028	1776	1646	1386
C	2415	2653	2184	1709	1867	1478
AT	2812	2485	2304	2611	2245	2086
BT	2697	2507	2306	408	355	321
PT	2746	2332	2250	1972	1578	1535
CT	2685	2685	3429	1911	1826	2300

**Table 9:** Glass transition temperatures and area under tanδ for all compositions.

Sample	*T*_g_ (°C)			Area under tanδ		
	Solid	ScF 1	ScF 2	Solid	ScF 1	ScF 2
A	75.1	71.1	71.2	27.3	26.1	24.9
B	−27.4	−27.55	−27.35	9	9.9	10.9
P	72.9	71.7	71.8	11.6	12.17	12.35
C	64.5	64	63.7	12	13.5	13.4
AT	72	72.5	72.3	26.4	23.2	25.3
BT	−28.3	−28.7	−28.9	8.8	8.3	8.1
PT	71.2	71.4	71.8	12.48	12.9	12.9
CT	64.2	64	64	13.7	8.1	14.4
